# Supplementary material for: The Influence of Herbicides to Marine Organisms Aliivibrio fischeri and Artemia salina
Source: Toxics. 2021 Oct 21;9(11):275. doi: 10.3390/toxics9110275 (PMC8623538; doi:10.3390/toxics9110275)
Supplement: Supplementary file 1 [file toxics-09-00275-s001.zip › toxics-1332590-supplementary-updated.pdf]

toxics

MDPI

Supplementary Materials: The Influence of Herbicides to Marine Organisms *Aliivibrio fischeri* and *Artemia salina*

Radek Vurm, Lucia Tajnaiová and Jana Kofroňová

1. Tested concentrations

Table S1. Luminiscent bacteria test. Tested concentrations of herbicides and active substances.

		Tested concentrations (μg.l⁻¹)
Herbicides	Roundup® Classic Pro	500; 400; 350; 300; 250; 200
	Kaput® Premium	6000; 5000; 4000; 3000; 2500; 2000; 1000
	Banvel® 480 S	10,000; 8750; 7500; 6250; 5000; 3750; 2500; 2000; 1500; 1000
	Lontrel 300	40,000; 30,000; 20,000; 10,000; 7500; 5000; 2500
	Finalsan®	750; 500; 300; 100; 70; 37.5; 10
Active substances	glyphosate	30,000; 25,000; 21,000; 14,000; 9000; 3500
	dicamba	39,500; 34,500; 26,000; 19,500; 9500; 5000
	clopyralid	31,000; 25,000; 19,500; 14,500; 9500; 5000
	nonanoic acid	75,000; 50,000; 25,000; 17,500; 10,000; 5000

Table S2. Crustacea bioassay test. Tested concentrations of herbicides and active substances.

		Tested concentrations (μg.l⁻¹)
Herbicides	Roundup® Classic Pro	50; 28; 22; 18; 14
	Kaput® Premium	40; 30; 20; 15; 10
	Banvel® 480 S	5000; 3700; 2500; 2000; 500
	Lontrel 300	5000; 3000; 2000; 1500; 1000; 500
	Finalsan®	1000; 300; 100; 70; 37; 10
Active substances	glyphosate	2000; 1000; 800; 600; 500
	dicamba	7500; 5000; 4500; 4000; 2000
	clopyralid	3000; 2800; 2600; 2500; 2000
	nonanoic acid	10,000; 9000; 7500; 7000; 6000; 5000

2. Curve fitting and probit curve fitting values.

Table S3. Tested organism *A. fischeri*.

Tested substances	Time (min)	Curve fitting							Probit curve fitting		
		Slope	Intercept	SD (σ)	SE	Chi-test (χ²) Sig	df	R^2	Slope	Intercept	R^2
Roundup® Classic Pro	15	4.0505	−4.6163	0.2469	0.0438	0.9637	4	0.9416	4.0050	−4.5085	0.9391
	30	4.5889	−5.9460	0.2179	0.0392	0.9043	4	0.9252	4.5137	−5.7680	0.9210
Kaput® Premium	15	2.7016	−4.1691	0.3701	0.0601	0.9585	5	0.9760	2.6821	−4.1020	0.9764
	30	2.8386	−4.6922	0.3523	0.0575	0.9851	5	0.9809	2.8198	−4.6288	0.9818
Banvel® 480 S	15	1.7341	−0.9325	0.5767	0.0774	1.0000	8	0.9955	1.7334	−0.9302	0.9955
	30	1.8047	−1.0626	0.5541	0.0757	1.0000	8	0.9945	1.8029	−1.0561	0.9946
Lontrel 300	15	1.8160	−2.0479	0.5507	0.0916	0.9852	5	0.9890	1.8110	−2.0278	0.9890
	30	2.0445	−3.0597	0.4891	0.0827	0.9672	5	0.9892	2.0379	−3.0324	0.9890
Finalsan®	15	1.0444	3.1081	0.9574	0.1559	0.9833	5	0.9876	1.0423	3.1130	0.9877
	30	0.9349	3.2957	1.0696	0.1713	0.9989	5	0.9943	0.9338	3.2980	0.9943
glyphosate	15	1.0363	0.9599	0.9650	0.1610	0.8902	4	0.8020	1.0156	1.0396	0.8079

	30	1.1444	1.0217	0.8738	0.1602	0.9566	4	0.7748	1.1031	1.1761	0.7924
dicamba	15	1.7585	−2.3871	0.5687	0.0974	0.8803	4	0.9361	1.7381	−2.3044	0.9392
	30	1.5620	−1.1908	0.6402	0.1122	0.9183	4	0.8736	1.5253	−1.0476	0.8742
clopyralid	15	1.7315	−1.9565	0.5775	0.0983	0.8501	4	0.9004	1.7019	−1.8379	0.9030
	30	1.7142	−1.3675	0.5834	0.1085	0.9727	4	0.8174	1.6263	−1.0258	0.8341
nonanoic acid	15	1.1107	0.3295	0.9004	0.1513	0.9897	4	0.9820	1.1102	0.3315	0.9821
	30	1.2078	−0.0095	0.8280	0.1406	0.9089	4	0.9532	1.2024	0.0132	0.9533

SD: standard deviation, SE: standard error, Chi-test: Chi-Square test, df: degree of freedom.

Table S4. Tested organism *A. salina*.

Tested substances	Curve fitting							Probit curve fitting		
	Slope	Intercept	SD (σ)	SE	R^2	Chi-test (χ^2) Sig	df	Slope	Intercept	R^2
Roundup® Classic Pro	7.0390	−3.9130	0.1420	0.0310	0.9999	1.0000	2	7.0392	−3.9129	0.9980
Kaput® Premium	6.5443	−3.5099	0.1528	0.0361	0.9893	0.9676	2	6.5128	−3.4709	0.9881
Banvel® 480 S	4.8253	−11.4114	0.2072	0.0421	0.9514	0.9221	2	4.7823	−11.2659	0.9699
Lontrel 300	2.5485	−3.2962	0.3924	0.0709	0.9612	0.9715	3	2.5263	−3.2214	0.9535
Finalsan®	1.6313	1.7147	0.6130	0.1169	0.8877	0.8396	4	1.5784	1.8425	0.8983
glyphosate	11.2942	−27.8573	0.0885	0.0236	0.9099	0.4585	1	11.0164	−27.0468	0.9889
dicamba	3.5048	−7.5046	0.2853	0.0626	0.9557	0.4448	2	3.4912	−7.4596	0.9381
clopyralid	9.8481	−28.9533	0.1015	0.0194	0.7701	0.9090	3	9.6924	−28.4121	0.7948
nonanoic acid	5.9059	−17.8837	0.1693	0.0292	0.8751	0.7836	1	5.7527	−17.2902	0.8775

SD: standard deviation, SE: standard error, Chi-test: Chi-Square test, df: degree of freedom
